# Supplementary material for: The Systems Biology Research Tool: evolvable open-source software
Source: BMC Syst Biol. 2008 Jun 29;2:55. doi: 10.1186/1752-0509-2-55 (PMC2446383; doi:10.1186/1752-0509-2-55)
Supplement: Additional file 1 — SBRT Archive. An archive of the current version of the Systems Biology Research Tool. [file 1752-0509-2-55-S1.zip › sbrt-1.4.0/doc/developers_guide/api/sbrt/shell/io/package-summary.html]

sbrt.shell.io


|  |  |  |
| --- | --- | --- |
| **Overview** \| **Package** \| Class \| **Use** \| **Tree** \| **Deprecated** \| **Index** \| **Help** | |  |
| **PREV PACKAGE**   **NEXT PACKAGE** | **FRAMES**    **NO FRAMES**     **All Classes** |  |


---

## Package sbrt.shell.io

Provides classes and interfaces for reading and writing files.

**See:**
  
          **Description**

| **Interface Summary** | |
| --- | --- |
| **InputFile<E>** | This interface is used to represent input files. |
| **MapInputFile<K,V>** | This interface is used to represent input files that contain maps. |
| **MapOutputFile<K,V>** | This interface is used to represent output files that maps can be written to. |
| **MapTextFile<K,V>** | This interface is used to represent text-based files that contain maps. |
| **MapTextInputFile<K,V>** | This interface is used to represent text-based input files that contain maps. |
| **MapTextOutputFile<K,V>** | This interface is used to represent output files that maps can be written to as text. |
| **OutputFile<T>** | This interface is used to represent output files. |
| **SbrtFile** | This interface is used to represent files. |

| **Class Summary** | |
| --- | --- |
| **AbstractMapInputFile<K,V>** | This abstract class is a skeleton implemenation of text-based map input files. |
| **AbstractMapOutputFile<K,V>** | This abstract class is a skeleton implementation of `MapTextOutputFile`. |
| **AbstractSbrtFile** | This class provides a helper method for SBRT files. |
| **ComiFile<K,V>** | This class is used to read column-oriented map input files. |
| **ComiFileSource<K,V>** | This class is used to provided a source of column-oriented map input files. |
| **ComoFile<K,V>** | This class is used to write maps to text files in a column-oriented way. |
| **ComoFileSource<K,V>** | This class is used to provided a source of column-oriented map output files. |
| **DevNull** | This class is used to mimic /dev/null from Unix-like operating systems. |
| **EndOfFile** | This class is used to represent end-of-file (EOF). |
| **FileFormat** | This class is used to represent file formats. |
| **FileLineIterator<E>** | This class is used to iterate through the lines of a file. |
| **MultiOutputFiles<F extends OutputFile<R>,R>** | This class is used to create multiple output files. |
| **ProcessInputFile** | This class is used to represent process input files. |
| **RomiFile<K,V>** | This class is used to read row-oriented map input files. |
| **RomiFileSource<K,V>** | This class is used to provided a source of row-oriented map input files. |
| **RomoFile<K,V>** | This class is used to write maps to text files in a row-oriented way. |
| **RomoFileSource<K,V>** | This class is used to provided a source of row-oriented map output files. |
| **SerializedFileIterator<E>** | This class is used to iterate over the objects in a serialized input file. |
| **SerializedInputFile<E>** | This class is used to read input files containing serialized objects. |
| **SerializedMapInputFile<K,V>** | This class is used to read input files containing serialized maps. |
| **SerializedOutputFile<E>** | This class is used to write serialized objects to output files. |
| **SmoFile<K,V>** | This class is used to write serialized maps to output files. |
| **SmoFileSource<K,V>** | This class is used to provided a source of serialized map output files. |
| **TextFileLineIterator<E>** | This class is used to iterate over the lines of an input file. |
| **TextInputFile<E>** | This class is used to read input files containing text. |
| **TextOutputFile<T>** | This class is used to represent output files containing plain text. |
| **WorkingDirectory** | This class is used to manage the working directory of the SBRT. |
| **XmlFileFactory** | This class is used to create DOM objects from XML files. |

## Package sbrt.shell.io Description

Provides classes and interfaces for reading and writing files.

---


|  |  |  |
| --- | --- | --- |
| **Overview** \| **Package** \| Class \| **Use** \| **Tree** \| **Deprecated** \| **Index** \| **Help** | |  |
| **PREV PACKAGE**   **NEXT PACKAGE** | **FRAMES**    **NO FRAMES**     **All Classes** |  |


---
